# Conservation of griseofulvin genes in the *gsf* gene cluster among fungal genomes

**DOI:** 10.1093/g3journal/jkab399

**Published:** 2021-11-18

**Authors:** Parisa Aris, Lihong Yan, Yulong Wei, Ying Chang, Bihong Shi, Xuhua Xia

**Affiliations:** Department of Biology, University of Ottawa, Ottawa, ON K1N 6N5, Canada; National Joint Engineering Research Center of Industrial Microbiology and Fermentation Technology, College of Life Sciences, Fujian Normal University, Fuzhou 350117, China; Department of Bioengineering, College of Life Sciences, Fujian Normal University, Fuzhou, China; Department of Biology, University of Ottawa, Ottawa, ON K1N 6N5, Canada; National Joint Engineering Research Center of Industrial Microbiology and Fermentation Technology, College of Life Sciences, Fujian Normal University, Fuzhou 350117, China; Department of Bioengineering, College of Life Sciences, Fujian Normal University, Fuzhou, China; National Joint Engineering Research Center of Industrial Microbiology and Fermentation Technology, College of Life Sciences, Fujian Normal University, Fuzhou 350117, China; Department of Bioengineering, College of Life Sciences, Fujian Normal University, Fuzhou, China; Department of Biology, University of Ottawa, Ottawa, ON K1N 6N5, Canada; Ottawa Institute of Systems Biology, Ottawa, ON K1H 8M5, Canada

**Keywords:** griseofulvin, *gsf* gene cluster, *Penicillium griseofulvum*, polyketide compound

## Abstract

The polyketide griseofulvin is a natural antifungal compound and research in griseofulvin has been key in establishing our current understanding of polyketide biosynthesis. Nevertheless, the griseofulvin *gsf* biosynthetic gene cluster (BGC) remains poorly understood in most fungal species, including *Penicillium griseofulvum* where griseofulvin was first isolated. To elucidate essential genes involved in griseofulvin biosynthesis, we performed third-generation sequencing to obtain the genome of *P. griseofulvum* strain D-756. Furthermore, we gathered publicly available genome of 11 other fungal species in which *gsf* gene cluster was identified. In a comparative genome analysis, we annotated and compared the *gsf* BGC of all 12 fungal genomes. Our findings show no gene rearrangements at the *gsf* BGC. Furthermore, seven *gsf* genes are conserved by most genomes surveyed whereas the remaining six were poorly conserved. This study provides new insights into differences between *gsf* BGC and suggests that seven *gsf* genes are essential in griseofulvin production.

## Introduction

Griseofulvin (C_17_H_17_Cl_1_O_6_) is a natural spirocyclic polyketide compound that is produced by ascomycetes. This fungistatic was first isolated from *Penicillium griseofulvum* Dierckx (syn. *Penicillium patulum* Bain.; *Penicillium urticae* Bain.) and had since been detected in many other species of the *Penicillium* genus such as *Penicillium aethiopicum* (Chooi *et al.* 2010). Griseofulvin synthesis increases with cellular ATP/ADP ratio (Rogal and Malkov 1978; Dasu and Panda 1999), and one may hypothesize that the benefit of griseofulvin production is to eliminate other fungal species to reduce competition. Nevertheless, given the cost of time and energy, one expects that only species that experience such competition would maintain the griseofulvin gene cluster in their genomes. Indeed, fungal endophytes such as *Xylaria flabelliformis* are commonly found within plant tissue and produces griseofulvin as an antifungal against plant pathogenic fungi (Whalley 1996). Furthermore, species in Genus *Penicillium* produce many other secondary metabolites in response to environmental changes, and some exometabolites are only expressed under unique circumstances (Frisvad 2014).

Mechanistically, griseofulvin interacts with the mitotic spindle microtubule to inhibits cell division and induces cell death in cancer cell lines (Rebacz *et al.* 2007). Due to its low toxicity (Hamdy *et al.* 2017), Griseofulvin has been widely used in many applications. In agriculture, griseofulvin acts as a crop protectant to prevent against fungus infections (Dayan *et al.* 2009). Notable medical applications of griseofulvin include treatment of ringworm infection in guinea pigs (Gentles 1958) and treatment of dermatophyte infections in both humans and animals (Oxford *et al.* 1939; Lambert *et al.* 1989). Today, griseofulvin is largely replaced by new antifungal drugs, but the compound retained a niche in treating ringworm infection and athlete’s foot (Chooi *et al.* 2010). In addition, in recent years, the compound has garnered renewed interest because of its potential therapeutic effects to treat cancer (Rebacz *et al.* 2007) and suppressive activities against the replication of hepatitis C virus (Jin *et al.* 2008).

Griseofulvin is synthesized by nonreducing polyketide synthases (NR-PKSs). To optimize the bioengineering of this natural compound for use in veterinary and medical applications, there have been continuous efforts to determine the *gsf* synthase genes and the griseofulvin synthetic pathway. While early degradation (Birch *et al.* 1958) and isotope labeling (Harris *et al.* 1976; Simpson and Holker 1977) experiments have determined that the biosynthetic process of griseofulvin involves several synthases to convert Acetyl-CoA and Malonyl-CoA (Birch and Donovan 1953; Birch *et al.* 1958; Simpson and Holker 1977) into a grisan scaffold (Kaufman and Sindelar 1989), it was not until 2010 that the *gsf* biosynthetic gene cluster (BGC) that is responsible for griseofulvin biosynthesis was fully discovered.

Through shotgun sequencing and Bioinformatics mining for PKS genes, *gsfA—gsfK*, *gsfR1*, and *gsfR2* were identified in the *gsf* BGC in *P. aethiopicum* (Chooi *et al.* 2010). The involvement of these *gsf* genes in griseofulvin biosynthesis was later determined through single-gene deletion and biochemical assay experiments (Cacho *et al.* 2013). To summarize the findings of Cacho *et al.* (2013), *gsfA* initiates the biosynthesis of griseofulvin; it is a nonreducing polyketide synthase that combines one Acetyl-CoA and six Malonyl-CoA units to generate the heptaketide backbone benzophenone 5a. Next, the O-methyltransferases (*gsfB* and *gsfC*) methylate phenols on benzophenone 5a to generate the intermediate griseophenone C. Following this process, griseophenone C is chlorinated and converted into griseophenone B by the halogenase *gsfI*, and griseophenone B is then converted into the grisan core by the phenol oxidative activity of *gsf F.* Finally, the grisan core is converted into the final griseofulvin through two additional steps: methylation at 5-OH catalyzed by *gsf D* and enoylredcution by dehydrogenase *gsfE*.

While some *gsf* genes are important in griseofulvin biosynthesis, others may not be essential. For instance, both *gsfA* and *gsfI* play crucial roles in the *gsf* biosynthetic pathway, and their deletions disrupt griseofulvin synthesis (Chooi *et al.* 2010; Cacho *et al.* 2013; Banani *et al.* 2016). In contrast, deletion of *gsfK* did not affect biosynthesis in *P. aethiopicum* (Cacho *et al.* 2013). A simple comparison between two griseofulvin-producing species, *P. aethiopicum* (GenBank accession ID GU574478.1) and *P*. *griseofulvum* PG3 (GenBank accession ID LHQR00000000.1), shows that *P*. *griseofulvum* lacks *gsfK*, *gsfH*, and *gsfR2* in the *gsf* BGC (Banani *et al.* 2016). Nonetheless, while experimental studies have detected griseofulvin production in many *Penicillium* species (Clarke and Mckenzie 1967; Frisvad and Samson 2004; Jadulco *et al.* 2004; Petit *et al.* 2004; Wang *et al.* 2004; Larsen *et al.* 2005; Xue *et al.* 2006; Chooi *et al.* 2010; Samson and Pitt 2013; Tang *et al.* 2015; Lee *et al.* 2016; Roullier *et al.* 2016; Nielsen *et al.* 2017; Ribeiro *et al.* 2018; Mead et al. 2019) and in species such as *X.*  *flabelliformis*, *Abieticola koreana*, and *Stachybotrys levispora* (Lee *et al.* 2016; Ribeiro *et al.* 2018; Mead et al. 2019), the biosynthetic details of the *gsf* BGC are scarce for most species.

We sequenced the genome of a biochemically unique *P. griseofulvum* strain D-756. It is a mutant strain with a high yield of griseofulvin originally isolated from a wild strain *P. patulum* 4541 (*patulum* is synonym of *griseofuvum*) and subject to multiple generations of mutagenesis (Wu et al. 1980). Strain D-756 features high tolerance of Cl^-^ which is an ingredient in griseofulvin synthesis. In particular, it can use starch as a carbon source instead of lactose in the original strain. We compared the *gsf* BGC between *P. griseofulvum* strain D-756 and 11 other fungal species that are known to produce griseofulvin. We found that 7 out of 13 identified *gsf* genes are well conserved among 12 griseofulvin-producing fungal species. Optimizing griseofulvin synthesis requires the identification of a set of essential *gsf* genes that are highly conserved in griseofulvin-producing fungi. However, there would be less evolutionary advantage to maintain nonessential *gsf* genes, and we expect these *gsf* genes to accumulate high sequence variability or are lost entirely at the *gsf* BGC of many fungal genomes.

## Materials and methods

### Strain

*Penicillium griseofulvum* Dierckx (syn.: *P. patulum* Bainier.; *P. urticae* Bainier) D-756 was a mutant strain with high yield of griseofulvin, which was obtained from the wild strain *P. patulum* 4541 after 13 rounds of artificial mutagenesis (Wu *et al.* 1980). The *P. griseofulvum* D-756 and its parental strain *P. griseofulvum* 4541 (syn.: *P. patulum* Bainier) were identified based on morphology and DNA sequencing of ITS and 18S rRNA (Lu *et al.* 2019).

### Third-generation sequencing on *Penicillium griseofulvum* strain D-756

The genome of *P. griseofulvum* strain D-756 was sequenced using the 3rd generation sequencing approach. First, genomic DNA from *P.*  *griseofulvum* D-756 was prepared as described previously by Möller et al. (1992). Briefly, spore suspensions of *P. griseofulvum* D-756 (1.0 × 10^7^ con/ml) were inoculated on a 25 ml solid Czapek-Dox Medium (CDM) plate and incubated at 28°C for 4 days. Fungal hyphae were collected and quickly grinding in a mortar with liquid nitrogen. DNA was extracted from 0.5 g frozen mycelia. DNA concentration and purity were analyzed by ultrafine spectrophotometer (DeNovix DS-11), and the DNA integrity was checked by agarose gel electrophoresis. The genome of strain D-756 was sequenced by Single Molecule Real-Time (SMRT) technology. Sequencing was performed at the Beijing Novogene Bioinformatics Technology Co., Ltd. Low-quality reads were filtered by the SMRT Link v5.0.1, and the filtered reads were assembled to generate contigs without gaps.

### Retrieving fungal genomes and their griseofulvin genes

We retrieved known Gsf protein sequences in the reference species *P. aethiopicum* (GenBank accession ID GU574478) as queries in BLASTP. The BLASTP searches were performed using the National Center for Biotechnology Information (NCBI) Nucleotide BLAST program (www.ncbi.nlm.nih.gov/nucleotide) with default options to identify fungal species that may harbor genes involved in griseofulvin synthesis. The top hundred hits for each Gsf proteins (GsfA-GsfR2) were represented in Supplementary File S1. The search results revealed 22 species that each contains at least three homologs with more than 50% BLASTP similarities to reference Gsf proteins in *P.*  *aethiopicum* (Supplementary File S1 and Table S1). Moreover, some species were previously reported to produce griseofulvin with experimental evidence (Supplementary File S2 and Data S1, for reports of fungi with griseofulvin production). The genome of these species was retrieved from the NCBI genome database (https://www.ncbi.nlm.nih.gov/genome). We also included more than 200 species from different genus selected from phylum Ascomycetes. In total, the genomes of 266 fungal species were retrieved from the NCBI database for further analysis (Supplementary File S1 and Table S2).

Among retrieved genomes were that of two *P.*  *griseofulvum*, strain PG3 and strain MRI314, under the Genome assembly accession GCA_001561935.1 and GCA_001735785.1, respectively. They were sequenced with Illumina MiSeq with an assembly level of sequence contig. While the International Sequence Database Collaboration (INSDC) submitter provided annotation for strain PG3, there is no annotation information for strain MRI314. To better annotate the *gsf* BGC in *P. griseofulvum*, we performed third-generation sequencing on *P. griseofulvum* strain D-756 (see above section) to identify and annotate its *gsf* BGC.

### Annotating and comparing the *gsf* BGC between fungal species

The fungal genomes were analyzed using Antibiotic Secondary Metabolite Analysis Shell (antiSMASH), a genome mining tool that integrates NCBI BLAST+, HMMer 3, Muscle 3, and FastTree to predict, identify, and perform secondary analysis of BGCs (Blin *et al.* 2019). The 266 genomes (Supplementary File S1 and Table S2), including the annotated genome of *P. griseofulvum* strain D-756 using funannotate (v1.5.1) (Love *et al*. 2018), were processed via antiSMASH v6.0 using the ClusterFinder algorithm with default settings to predict gene cluster borders and to annotate predicted griseofulvin gene cluster. The Minimum Information about a Biosynthetic Gene cluster (MIBiG) repository provides reference gene clusters as a framework for the classification of BGCs (Kautsar *et al.* 2020), and MIBiG accession BGC0000070 (the griseofulvin BGC from *P. aethiopicum*) was used as a reference cluster in this study to find the griseofulvin gene cluster in fungal genomes. Nucleotide sequences of interest identified by antiSMASH were used to perform BLASTN against *P. aethiopicum* IBT 5753 (GenBank accession ID GU574478) as query to test consistency of predicted *gsf* genes.

The antiSMASH outputs were analyzed using Biosynthetic Gene Similarity Clustering And Prospecting Engine (BiG-SCAPE), a software package that explores the diversity of BGCs across genomes (Navarro-Muñoz *et al.* 2020). Briefly, BiG-SCAPE determines the functional protein domains of the biosynthetic griseofulvin gene cluster for all species from the Pfam database (https://pfam.xfam.org/). Then, every predicted protein domain was aligned using hmmalign, which is a part of the HMMer package that used profile hidden Markov models (HMMs) for biological sequence analysis. In addition, the pairwise distance between gene clusters was calculated using a combination of three indices, including Jaccard Index (JI), the Adjacency Index (AI), and the Domain Sequence Similarity (DSS). The sequence similarity networks of BGCs, directly from antiSMASH results and MIBiG reference gene clusters, were constructed based on a comparison of their protein domain content, order, copy number, and sequence identity (Navarro-Muñoz *et al.* 2020). Following BiG-SCAPE, CORASON provided the phylogenetic relationships of these griseofulvin BGCs using FastTree (default options) with the Jukes-Cantor + CAT model for a nucleotide alignment.

Pfam protein families (http://pfam.xfam.org) and the NCBI conserved domain database (CDD) (https://www.ncbi.nlm.nih.gov/cdd/) were used for interactive domain family analysis of *gsf* BGC in fungal species.

### Phylogenetic reconstruction

Gsf protein sequences from antiSMASH results were first aligned using MAFFT with the slow but accurate G-INS-i option (Katoh *et al.* 2009) and aligned genes were concatenated in order. The phylogenetic tree was then constructed using PhyML (Guindon and Gascuel 2003) with the GTR model which is the best-fitting model for the dataset based on likelihood ratio tests and information-theoretic indices such as AIC and BIC (Akaike 1998). Moreover, FastME with the LG (Le and Gascuel 2008) substitution model was used for phylogenetic inference based on distance methods in order to confirm the analysis. *Memnoniella*  *echinata* was selected as the outgroup, and bootstrapping tests were performed with 500 repetitions.

The ITSx program (https://microbiology.se/software/itsx/), a Perl-based software tool that implements hidden Markov models, was used to predict ITS region in *P. griseofulvum* D-756 based on predicted positions of the ribosomal genes (Bengtsson‐Palme et al. 2013). The ITS sequences of other species were retrieved from NCBI Fungal ITS RefSeq Targeted Loci Project (accession PRJNA177353). These sequences were then aligned using MAFFT with G-INS-i option, and phylogenetic trees were constructed in DAMBE (Xia 2018) using PHYML, with GTR as the best model based on AIC, bootstrap = 500, and outgroup species = *X. flabelliformis*.

## Results and discussion

The biosynthesis pathway of griseofulvin has been previously elucidated in *P. aethiopicum* strain IBT 5753 (Chooi *et al.* 2010). A total of 13 genes were identified in the *gsf* BGC, including nonreducing polyketide synthase (NR-PKS), tailoring enzymes, dehydrogenase/reductase, cytochrome P450, halogenase, ketoreductase, and transcription factors (Chooi *et al.* 2010). In the present study, griseofulvin BGCs were predicted in 11 other fungal species: *Penicillium capsulatum*, *Penicillium coprophilum*, *Penicillium vulpinum*, three *P.*  *griseofulvum*, two *Aspergillus alliaceus*, *Aspergillus burnettii*, *X.*  *flabelliformis*, and *M.*  *echinata*, which showed homology to MIBiG accession BGC0000070, the reference griseofulvin BGC from *P. aethiopicum* (Supplementary File S2 and Data S2). Of note are *M. echinata* and *X. flabelliformis*, two species that were previous reported to synthesize griseofulvin, yet the genes involved in the biosynthetic pathway were unknown (Jarvis *et al.* 1996; Mead et al. 2019).

The antiSMASH result of *P. griseofulvum* D-756 showed the *gsf* gene cluster is located at the genomic positions 1364595–1389034 contig 4 (Supplementary File S3 and Table S10). The BGC of *P. griseofulvum* D-756 contains the same *gsf* genes with more than 99% homology and organized in the same order as those in the BGC of *P. griseofulvum* strain PG3 (Supplementary File S2 and Data S3). Expectedly, the *gsf* genes are conserved between these two strains except *gsfG*. In addition, the griseofulvin BGC was annotated in the other nine species (Supplementary File S3 and Tables S1–S11) belong to the Aspergillaceaea family of the order Eurotiales, also known as the green and blue molds, and 2 species, *M. echinata* and *X. flabelliformis*, belonging to the Hypocreales order and Xylariales order, respectively (Supplementary File S1 and Table S2). Species belonging to the Aspergillaceaea family exhibit diverse ecology. For example, genus *Penicillium* have been frequently isolated from different environmental sources such as air, freshwaters, terrestrial, food product, and soil (Zhang and Wang 2015), potentially due to their low nutritional requirements and large enzymatic apparatus for primary metabolism (Cruz *et al.* 2013). Among these species, *Aspergillus alliaceus* has effective biological control of the root parasitic weed (Aybeke *et al.* 2014). *Xylaria*  *flabelliformis* is another griseofulvin producing species which is commonly found within plant tissue. These species are usually produce griseofulvin to control other fungi and it allows them to decompose of cellulose in the senescent plant (Whalley 1996; Mead *et al*. 2019). This indicates griseofulvin can be produced by a variety of fungi and not limited to *Penicillium* species. However, our results indicate that the *gsf* BGC is only conserved among some species in the Eurotiomycetes class and the Sordariomycetes class (Supplementary File S1 and Table S2). In addition to the presence of *gsf* BGC in the genome, several external factors are also involved in producing griseofulvin including organic materials, such as source of carbon, levels of ATP and nitrogen (Wright 1955; Soloveva and Malkov 1972). For example, using glucose or acetate as the source of carbon may generate the high level of ATP which leads to produce more griseofulvin (Alan *et al.* 1958; Dasu and Panda 1999). Another important factor is pH; griseofulvin yield increases at pH ranging from 5.5 to 6 (Alan *et al.* 1958).

**Figure 1:**
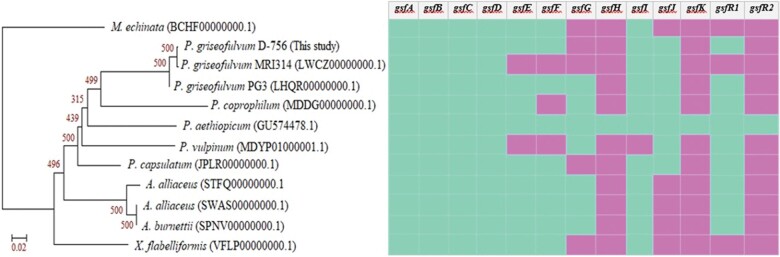
Phylogenetic reconstruction at the Gsf protein sequences in BGC of 12 species. The tree was built with the maximum likelihood-based PhyML approach, using aligned and concatenated Gsf protein sequences in BGC, with *M. echinata* as outgroup. Supporting bootstrap values are based on 500 replicates. The heat map on the right indicates the presence (blue) or absence (pink) of *gsf* genes identified in fungal genomes, against the *gsf* BGC of *P. aethiopicum* as reference, with rows representing species and columns representing *gsf* genes.

A maximum-likelihood phylogenetic analysis showed the global relationships among whole *gsf* BGCs of 12 surveyed fungal species. Figure 1 shows the three *P. griseofulvum* species clustered together in a clade very distant from *X. flabelliformis* and distinct from other members of the *A. alliaceus* group. Seven out of 12 species including *P. aethiopicum*, *X. flabelliformis*, *M. echinata*, *P. griseofulvum* strain PG3 and D-756, *P. vulpinum*, *P. coprophilum* are known to produce griseofulvin with experimental validations (Jarvis *et al.* 1996; Chooi *et al.* 2010; Banani *et al.* 2016; Nielsen *et al.* 2017; Mead et al. 2019). Another phylogenetic tree was generated by the FastME method in order to confirm the analysis (Supplementary File S2 and Figure S2). In addition, a reference phylogenetic tree was constructed using the ITS region for 48 species whose ITS sequences were available in NCBI or detected using the ITSx tool (Supplementary File S2 and Figure S3).

A nucleotide sequence comparison using BLAST (Supplementary File S3) at identified *gsf* genes, with reference being *P. aethiopicum*, revealed that many *gsf* genes are highly conserved (similarity >70%). These include nonreducing polyketide synthase (*gsfA*), tailoring enzymes (*gsfB*-*D*), dehydrogenase (*gsfE*), cytochrome P450 (*gsfF*), and halogenase (*gsfI*). However, the ankyrin repeats mediate protein-protein interactions (*gsfG*), drug resistance transporter (*gsfJ*), and transcription factor (*gsfR1*) are not well-conserved.

Moreover, we observed that amidohydrolases (*gsfH*), short chain dehydrogenase (*gsfK*), and Zn2Cys6 binuclear cluster domain (*gsfR2*) in *P. aethiopicum* were not located within *gsf* gene cluster in all other fungal genomes. However, the three *gsf* genes including *gsfH*, *gsfK* and *gsfR2* can be found by BLASTN in another region in the genome of *P. griseofulvum* strain D-756 (Supplementary File S4 and Table S1). This finding is similar to that previously reported in the genome of *P. griseofulvum* strain PG3, in which a homolog of *gsfR2* was identified outside of the *gsf* gene cluster (Banani *et al.* 2016). Hence, although these genes are not located within the *gsf* cluster, they may still be involved in griseofulvin production.

In brief, the above results revealed that seven strains harbor genes that encode the highly homologous proteins to Gsf BGC in the reference species *P. aethiopicum*. They are *P. griseofulvum* strain PG3 having ten *gsf* genes, followed by *P. griseofulvum* strain D-756, *P. capsulatum*, *P. coprophilum*, *A. burnettii*, and *A. alliaceus* (strain CBS 536.65 and strain IBT 14317) each having nine *gsf* genes. However, the BLASTN analysis against *P. aethiopicum* as query revealed greater number of griseofulvin genes in five fungal genomes, including *P. griseofulvum* strain D-756, *P. griseofulvum* strain MRI314, *P. vulpinum*, *P.coprophilum*, and *P. capsulatum*, compared to antiSMASH (Supplementary File S4 and Tables S1–S5). For example, although BLASTN hits show a homolog of *gsfG* located in the contig 4 of *P. griseofulvum* D-756 genome, that was not identified by antiSMASH prediction. However, this may be a false positive as the annotation shows this hit belongs to an uncharacterized protein. The Known Cluster Blast comparisons made by antiSMASH only show hits for genes with a percentage identity greater than 45% and minimum coverage of 40% (Blin *et al.* 2013). Anything below the cutoff points is not considered significant for the comparison. For some of the BLASTN results, some genes appear to be missing entirely in the annotated file, *e.g.*, *gsfG* in *P. capsulatum* genome. In this study, only the *gsf* genes identified by both antiSMASH and BLASTN were considered for further analysis.

Only seven *gsf* genes (*gsfA*-*gsfF*, *gsfI*) out of the 13 in *P. aethiopicum* of which were presented in more than 70% of species were conserved by other fungi, including close relative *Penicillium* species. This suggests that these seven *gsf* genes could be essential for griseofulvin production. That not all *gsf* genes may be essential is corroborated by a recent study Valente *et al.* (2020) showing that the *gsfR2* does not play a role in griseofulvin biosynthetic cluster and deletion of *gsfR1*, a negative regulator, led to an increase in griseofulvin production under specific conditions.

**Figure 2:**
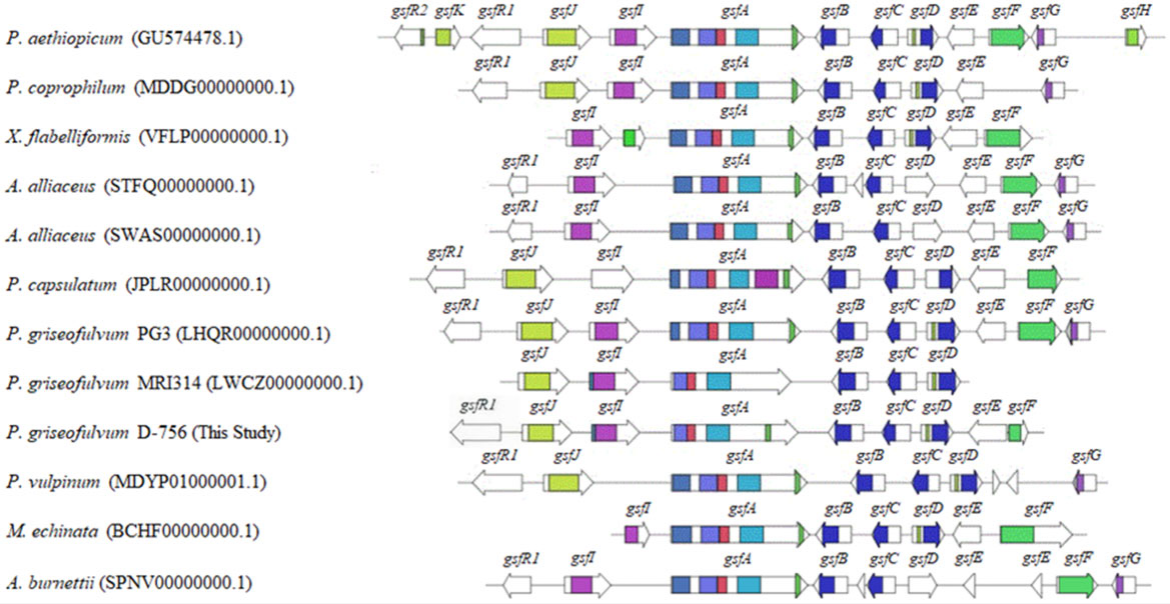
Organization of putative gene clusters involved in griseofulvin biosynthesis in surveyed fungal species. Arrows show the strand directionality of genes. The color codes within the genes denote the functional domains (for more information on Pfam domains, see the Supplementary File S2 and Data S4 and S5).

Locally, there are differences at *gsf* genes among fungal species, and our analysis at the *gsf* BGC revealed variable degrees of gene conservation that have not been reported previously (Figure 2). In the Pfam profiles (Supplementary File S2 and Data S4 and S5) and nucleotide sequences of *gsf* genes, we found differences in *gsf* genes that may lead to either loss of function (LOF) or gain of function (GOF). For example, BiG-SCAPE reveals that the GsfD (ADI24956.1) functional domains PF08100 (dimerization) and PF00891 (O-methyltransferase) do not exist in the *gsf* BGCs of either *A. alliaceus* stain IBT 14317 or strain CBS 536.65 (Figure 2). In addition, at GsfA (ADI24953.1), we found that all species shared three typical domains (PKS, PT, and ACP). However, while Pfam profile analysis revealed that *P. griseofulvum* strain D-756 and strain PG3 contain the GsfA domain PF00550 [phosphopantetheine attachment site (PP-binding)], *P. griseofulvum* strain MRI314 does not harbor this domain (Supplementary File S2 and Data S4 and S5). In addition, GsfA (ADI24953.1) in *P. capsulatum* has an extra PS-DH domain (PF14765, a polyketide synthase dehydratase) which catalyze dehydrations in the biosynthesis of some polyketides such as the antibiotic rifamycin and erythromycin (Keatinge-Clay 2008; Gay *et al.* 2013). Furthermore, at GsfI (ADI24948.1) and GsfD (ADI24956.1), although *P. capsulatum* harbors both *gsf* genes, the functional domains of GsfI (PF04820, a halogenase) and GsfD (PF08100, a dimerization domain) have been lost from the genes (Supplementary File S2 and Data S4). Further Gsf domain analysis was performed using NCBI CDD (Supplementary File S5 and Tables S1–12). CDD analysis revealed a 5beta-POR_like_SDR_domain (cd08948) in GsfE (ADI24957.1) among all surveyed species that encode homolog of GsfE. In addition, fungal_TF_MHR domain _which plays a regulatory role (Todd and Andrianopoulos 1997)_ identified in GsfR1 (ADI24950.1) among four *Penicillium* species including *P. griseofulvin* PG3, *P. capsulatum*, *P. coprophilum*, *P. aethiopicum*. However, it seems that this domain has been lost entirely from the genome of other species including *A. alliaceus*, *A. burnettii*, *X. flabelliformis*, and *M. echinata*. All of these are potential LOF or GOF events that potentially alter gene activity (Housden *et al.* 2017). Future Bioinformatics analyses and experimental research are required to examine and validate these potential LOF and GOF events.

**Figure 3:**
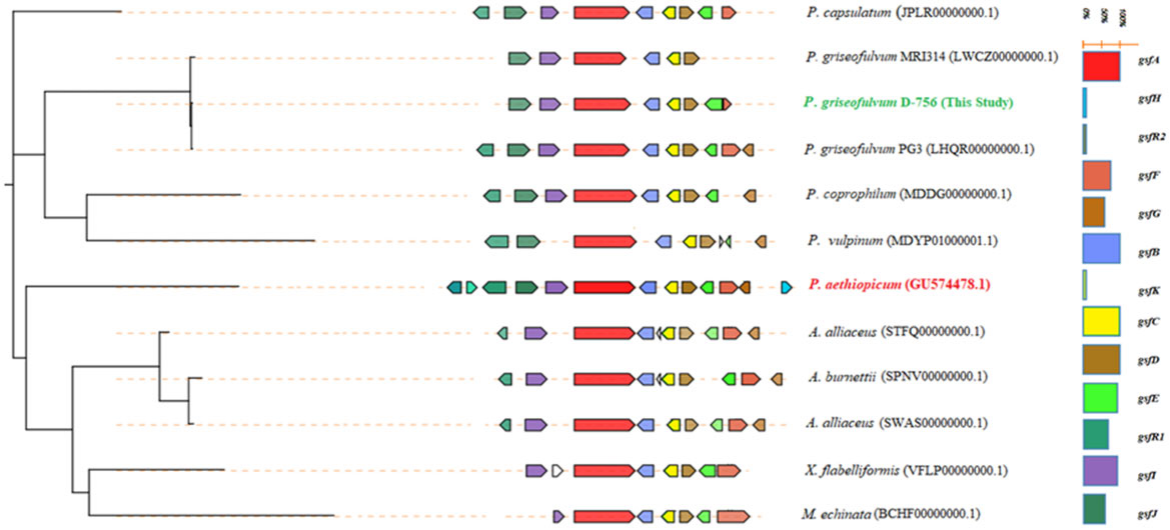
Phylogenetic tree of griseofulvin BGCs generated by CORASON with sequence similarities relative to the griseofulvin BGC in MIBiG (BGC000070), which is highlighted in red (*P. aethiopicum*). Highlighted in bold green (*P. griseofulvum* strain D-756) is sequenced in this study. The histogram on the right shows the name and color code for *gsf* genes and the level at the gene is conserved (in percentage) by the 12 species.

Finally, as a comparison of results between CORASON and BiG-SCAPE, Figure 3 shows that colored regions red and blue (representing the domain-containing genes *gsfA* and *gsfB*), region yellow and brown (representing the domain-containing genes *gsfC* and *gsfD*) are well conserved by all fungi species. This CORASON result is consistent with BiG-SCAPE results shown in Figure 2. For instance, genes *gsfE* and *gsfI* located at colored region green and purple are conserved in at least 10 out of 12 species, and Figure 3 shows that the core domain consisting of these *gsf* genes are indeed shared by more than 80% of species surveyed. In addition, the phylogenetic analysis of griseofulvin gene clusters in Figure 1, using sequences aligned by MAFFT and constructed based on the PhyML approach, is consistent with Figure 3, which was constructed using FastTree in CORASON. This finding shows an agreement between these two methods.

## Conclusion

This study provides genomic evidence to understand and identify *gsf* genes involved in griseofulvin biosynthesis among fungal species. We sequenced the genome of *P. griseofulvum* strain D-756 and retrieved available fungal genomes from the NCBI database. In total, we compared 11 *gsf* BGCs to the reference *gsf* BGC in *P. aethiopicum* with known biosynthetic pathway (Chooi *et al.* 2010; Cacho *et al.* 2013). Our results suggest that *gsfH*, *gsfK*, and *gsfR2* genes may not be essential for griseofulvin biosynthesis, consistent with findings from a previous study (Banani *et al.* 2016). Based on gene conservation between fungal species, a total of seven conserved *gsf* genes were determined, out of the 13 putative *gsf* cluster genes from *P. aethiopicum* (Chooi *et al.* 2010). Our results are consistent with those verified by Cacho *et al.* (2013). Furthermore, our finding also revealed that these seven *gsf* genes were not only maintained, but also have shared sequence orders, between fungal species. However, several differences in these *gsf* genes may lead to either LOF or GOF.

An interesting question involving these findings is that species that experience benefits for producing griseofulvin (*e.g.*, eliminating other fungal species to reduce competition) would maintain the griseofulvin gene cluster. If a species is freed from competition (*e.g.*, having evolved to exploit a new carbon source or having colonized an area without competing species, or in areas where resources are abundant), then the advantage of producing griseofulvin disappears and the gene cluster would likely degrade or evolve new functions. In addition, given that griseofulvin can interfere with mitosis and inhibit cell growth, another interesting question involving those griseofulvin-producing fungal species is their underlying detoxification pathway to protect themselves against griseofulvin. Future analyses to compare the genomes of griseofulvin-producing species and griseofulvin susceptible species could help identify the genes involved in the detoxification pathway.

## Data availability

This Whole Genome Shotgun project has been deposited at DDBJ/ENA/GenBank under the accession JAHTKM000000000, BioSample SAMN20034676. The version described in this study is version JAHTKM010000000. In addition, the whole genome sequence data (the genome assembly and annotation of *P. griseofulvum* D-756) have been deposited in Genome Warehouse in National Genomics Data Center, Beijing Institute of Genomics, Chinese Academy of Sciences/China National Center for Bioinformation, under accession number GWHBEUO00000000, belonging to the BioProject of PRJCA006461 that is publicly accessible at https://ngdc.cncb.ac.cn/gwh.

Supplementary material is available at *G3* online.

## Supplementary Material

jkab399_Supplementary_Data_files

jkab399_Supplementary_Dataset
